# Anesthetic Efficacy of Mental/Incisive Nerve Block Versus Inferior Alveolar Nerve Block in Mandibular Premolars With Symptomatic Irreversible Pulpitis: A Systematic Review and Meta-Analysis

**DOI:** 10.7759/cureus.96492

**Published:** 2025-11-10

**Authors:** Oscar Lozano González, Marco Felipe Salas Orozco, Jaime Trigueros Mancera, Noé Gustavo Martínez Cuellar, Carlos Luna Domínguez, Pedro Rosales García

**Affiliations:** 1 Dentistry, Clinical Research Laboratory, Faculty of Stomatology, Autonomous University of San Luis Potosí, San Luis Potosí, MEX; 2 Faculty of Stomatology, Autonomous University of San Luis Potosí, San Luis Potosí, MEX; 3 School of Dentistry, Universidad Latina de México, Guanajuato, MEX; 4 Graduate Program in Endodontics, University of Guadalajara, Guadalajara, MEX; 5 Graduate Program in Endodontics, Autonomous University of Tamaulipas, Tamaulipas, MEX; 6 Regional Complex of the Benemérita, Universidad Autónoma de Puebla, Puebla, MEX

**Keywords:** inferior alveolar nerve block, mandibular premolars, mental nerve block, meta-analysis, symptomatic irreversible pulpitis

## Abstract

This systematic review and meta-analysis aim to synthesize the current evidence from randomized clinical trials (RCTs) comparing the anesthetic success of mental/incisive nerve block (MINB) versus inferior alveolar nerve block (IANB) in patients with symptomatic irreversible pulpitis affecting mandibular premolars. A thorough literature search was performed using databases such as PubMed, Web of Science, Science Direct, Cochrane Database, and Google Scholar. The search covered publications up to June 2025. The focused PICOTT question for this review was: “What is the anesthetic efficacy of mental/incisive nerve block compared to inferior alveolar nerve block in mandibular premolars with symptomatic irreversible pulpitis?”. The included studies were evaluated for quality using the ROB 2 tool for non-randomized designs, while the certainty of the evidence was assessed through the GRADE approach. A meta-analysis was performed using a random-effects model to address potential variability among studies. Three RCTs were included. Overall, the risk of bias was considered low to moderate using the ROB 2 tool. The GRADE assessment indicated that the certainty of evidence was rated as very low. The overall pooled analysis showed no statistically significant difference in success rates between the MINB and IANB techniques (OR = 1.25; 95% CI: 0.74 to 2.12; P = 0.40), with low heterogeneity (I² = 0%). Subgroup analysis by premolar type showed no significant differences between techniques for either first (OR = 1.03; 95% CI: 0.64-1.65; P = 0.90; I² = 0%) or second premolars (OR = 1.23; 95% CI: 0.76-1.98; P = 0.40; I² = 0%). In conclusion, the findings of this systematic review and meta-analysis indicate no clear difference in anesthetic efficacy between MINB and IANB techniques. However, most studies suggest that achieving adequate pulpal anesthesia requires the use of supplemental anesthesia.

## Introduction and background

Achieving effective pulpal anesthesia in mandibular teeth remains a clinical challenge, especially in symptomatic irreversible pulpitis (SIP) cases. Several pathophysiological factors, such as the upregulation of tetrodotoxin-resistant sodium channels (TTX) and vanilloid receptors (TRPV1) and a rise in neuropeptides such as Substance P and calcitonin gene-related peptide, could reduce the effectiveness of local anesthetics in inflamed tissues [1].

While maxillary teeth often respond well to infiltration anesthesia, mandibular posterior teeth, particularly premolars and molars, are commonly anesthetized using the inferior alveolar nerve block (IANB) [2]. Although IANB is the standard technique, its reported success rate in SIP cases can be as low as 30-40%, necessitating the use of supplemental techniques such as intraosseous, intraligamentary, or buccal infiltrations [2-4]. Moreover, IANB carries risks including nerve injury, hematoma, needle breakage, and systemic toxicity due to vascular proximity [5,6].

The mental/incisive nerve block (MINB) is considered a more conservative alternative for anesthetizing mandibular premolars, canines, and incisors. It involves depositing an anesthetic in close proximity to the mental foramen (MF). Although MINB avoids many of the complications of IANB and offers a faster onset, it is associated with a shorter duration of anesthesia, absence of lingual nerve coverage, and occasional inadequate anesthesia requiring supplementation [7].

Both IANB and MINB require careful technique and clinical experience to achieve consistent anesthesia. The IANB involves identifying precise anatomical landmarks and placing the needle near the MF, while the MINB depends on accurate localization of the mental foramen to ensure effective anesthetic diffusion [8]. Anatomical variability in the mandibular canal and the position of the mental foramen can influence the success of both techniques. Because IANB targets the mandibular nerve before it enters the mandibular canal and MINB acts more distally, their distinct anatomical pathways may partially explain variations in clinical outcomes [9]. These differences highlight the importance of operator expertise and anatomical understanding in optimizing anesthetic predictability and safety [10,11].

Accurate localization of the MF is essential for the success of the MINB. Its position varies among individuals and populations, affecting anesthetic diffusion and success rates [12]. Cone-beam computed tomography provides precise visualization of the mental foramen and adjacent neurovascular structures, improving the accuracy of anesthetic deposition and minimizing potential complications [13,14].

Lidocaine and articaine are among the most commonly used anesthetic solutions in dental procedures. These agents differ in their chemical structure and pharmacodynamic properties. Lidocaine is an amide-type anesthetic with a well-established safety profile. In contrast, articaine is unique among amides due to its thiophene ring and ester group, which enhance its lipid solubility and allow for faster diffusion through soft and hard tissues [15]. Articaine is marketed at a 4% concentration because lower concentrations, such as 2%, have demonstrated inconsistent anesthetic efficacy, particularly in dense mandibular bone. This increased permeability makes articaine particularly effective for infiltration techniques, especially in dense mandibular bone [16].

Despite the clinical relevance of achieving profound anesthesia in mandibular premolars with SIP, current evidence regarding the superiority of MINB over IANB remains inconclusive. Some studies suggest that IANB achieves higher success rates in this context, particularly when combined with 4% articaine or supplemental techniques [10,17]. In contrast, other trials report comparable efficacy between MINB and IANB, especially when using articaine. Notably, Aggarwal et al. found that combining MINB with IANB resulted in significantly higher anesthetic success than either technique alone, suggesting a possible synergistic effect [18]. Additionally, several investigations found no significant difference between the two approaches when used independently [18,19].

Although prior meta-analyses have evaluated MINB versus IANB in broader mandibular populations [17,20], none have focused exclusively on mandibular premolars. This review therefore provides a premolar-specific synthesis of randomized trials in SIP. Understanding the comparative efficacy of these nerve blocks in premolars is essential because premolars present distinct anatomical and innervation characteristics compared to molars and anterior teeth. Moreover, identifying the predictable need for supplemental anesthesia with either technique could directly inform clinical decision-making and patient management in endodontic practice.

This systematic review and meta-analysis synthesize evidence from randomized clinical trials (RCTs) that evaluated patients with SIP in mandibular premolars, comparing the MINB with the IANB in terms of anesthetic success during endodontic procedures. By addressing this specific clinical scenario, the present review aims to provide a clearer understanding of the comparative efficacy of these techniques and offer practitioners more reliable evidence for optimizing anesthetic approaches in cases of SIP.

## Review

Materials and methods

Protocol and Registration

The study protocol was registered in the PROSPERO international prospective register of systematic reviews, CRD420251066879. This systematic review and meta-analysis used the Preferred Reporting Items for Systematic Reviews and Meta-Analyses (PRISMA) 2022 guidelines.

Eligibility Criteria

The focused PICOTT question for this review was: “What is the anesthetic efficacy of MINB compared to IANB premolars with SIP?” The eligibility criteria were as follows: Population: Adult patients diagnosed with SIP in mandibular premolars; Intervention: Mental/ incisive nerve block; Comparator: Inferior alveolar nerve block; Outcomes: Success of pulpal anesthesia (the absence of pain during endodontic access and instrumentation); Time: No restriction; Type of Study: Only RCTs were included. Studies had to provide sufficient data to extract dichotomous outcomes and be published in full text, regardless of language.

Studies were excluded if they did not specifically focus on IANB and MINB in the context of SIP in mandibular premolars. Additional exclusion criteria included opinion pieces, letters to the editor, narrative reviews lacking original data, case-control studies, case series, studies without a control group, and those with unclearly described methodologies for assessing anesthetic outcomes.

Search Strategy

A comprehensive electronic search was conducted across PubMed, Web of Science, ScienceDirect, Cochrane Database, and Google Scholar for studies published up to June 2025. The final searches were performed between June 14 and June 16, 2025. No restrictions were applied regarding language or publication date.

Boolean operators (“AND” and “OR”) were used in combination with Medical Subject Headings (MeSH) and title/abstract terms to maximize retrieval sensitivity. The primary PubMed search strategy was: (“Mental Nerve Block” OR “Mental/Incisive Nerve Block”) AND (“Inferior Alveolar Nerve Block”) AND (“Symptomatic Irreversible Pulpitis” OR “Irreversible Pulpitis” OR “Pulpitis” OR “Symptomatic”) AND (“Mandibular Premolars”).

The search strategy was appropriately adapted for each database to reflect indexing differences. Reference lists from the included articles were manually examined to find further eligible studies. The complete search strategies and the number of records retrieved from each database are detailed in Supplemental Table 1.

Study Selection

Two reviewers (O.L. and M.S.) independently carried out a comprehensive literature search across multiple electronic databases, including PubMed, Embase, Web of Science, Scopus, and ScienceDirect, up to June 2025. Google Scholar was also searched to ensure the inclusion of gray literature. No date restrictions were applied during the search process. All retrieved references were managed using EndNote X9 (Clarivate Analytics, New York, NY), and duplicates were systematically identified and eliminated prior to screening.

Two reviewers (O.L. and M.S.) independently reviewed the titles and abstracts of all retrieved studies to identify potentially eligible trials. Full-text versions of the selected articles were then assessed to determine final inclusion. Any reviewer discrepancies were resolved through discussion or, when necessary, by consulting a third reviewer (G.M.).

Primary and Secondary Outcomes

The primary endpoint was anesthetic success during endodontic access and instrumentation, defined as no pain or mild pain (e.g., NRS ≤3 or HP-VAS <55 mm) without supplemental anesthesia. Secondary endpoints (when reported) included onset time to pulpal anesthesia (minutes), need for supplemental anesthesia (yes/no), postoperative pain at early time points (e.g., 6-8 h and/or 24 h), soft-tissue/lip numbness (proxy for IANB), and adverse events.

Given potential misclassification between MINB and buccal infiltration that could bias comparisons with IANB, MINB was prospectively operationalized as injection after MF localization (palpation and/or imaging), with deposition toward the MF region. Trials without explicit MF localization were retained but coded a priori as “MINB without MF localization” for the planned subgroup. Pre-specified subgroups included tooth type (1st vs 2nd premolars), anesthetic solution (4% articaine vs 2% lidocaine), and MF localization in the MINB arm (Yes vs No). If any subgroup was not statistically estimable due to insufficient studies, results were presented descriptively. Additionally, leave-one-out sensitivity analyses were pre-specified to assess the robustness of findings.

Risk of Bias Assessment

The risk of bias for each included study was independently evaluated by two reviewers (O.L. and M.S.) using the Cochrane Risk of Bias 2.0 (RoB 2) tool, as all studies included in this review were randomized controlled trials. This assessment covered the following domains: randomization process, deviations from intended interventions, missing outcome data, measurement of the outcome, and selection of the reported result. Any disagreements were resolved through discussion or consulting a third reviewer (G.M.). Studies were classified as having “low risk” if all domains were judged to be at low risk, “some concerns” if at least one domain raised concerns, and “high risk” if one or more domains were rated as high risk of bias.

Quality of Evidence

The certainty of the evidence for each outcome was evaluated using the GRADE approach with the GRADEpro GDT software (McMaster University, Hamilton, ON, Canada). The assessment considered five domains: risk of bias, inconsistency of results, indirectness of evidence, imprecision, and potential for publication bias. Based on these criteria, each outcome was classified as having high, moderate, low, or very low certainty.

Data Synthesis

For the quantitative analysis, data from RCTs comparing the efficacy of MINB (intervention group) versus IANB (control group) were synthesized using odds ratios (OR) with 95% confidence intervals (CIs), as the outcome of interest was dichotomous (success vs. failure of pulpal anesthesia).

Meta-analyses were performed using a random-effects model, with between-study variance (τ²) estimated through the Restricted Maximum Likelihood (REML) method in RevMan (Version 5.3; Copenhagen: The Nordic Cochrane Centre, The Cochrane Collaboration, 2014), following the recommendations of the Cochrane Handbook for Systematic Reviews of Interventions (version 6.4, Section 10.10.4.1). The prediction interval (PI) was calculated to estimate the expected range of effects in future similar studies.

When appropriate, subgroup analyses were conducted to explore variability based on anesthetic solution or other study characteristics. Statistical heterogeneity among studies was assessed using the I² statistic, with values exceeding 50% indicating considerable heterogeneity. A p-value less than .05 was considered statistically significant. Publication bias was not evaluated due to the limited number of studies (n = 3) [21].

Results

Study Selection

**Figure 1:**
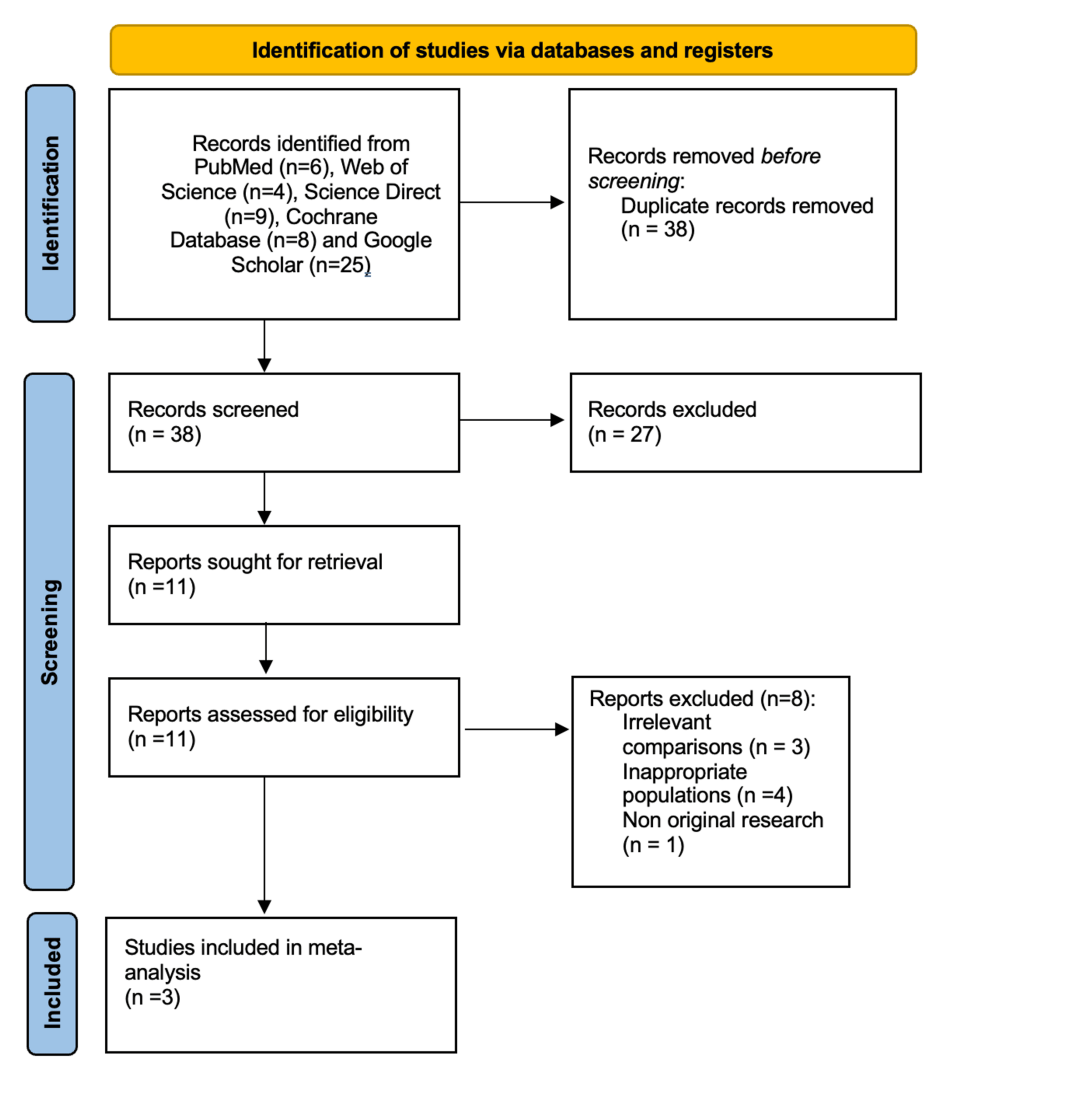
PRISMA flowchart of included studies PRISMA: Preferred Reporting Items for Systematic Reviews and Meta-Analyses

A total of 52 records were initially identified through searches in PubMed, Web of Science, ScienceDirect, the Cochrane Database, and Google Scholar. After removing 14 duplicates, 38 records remained for screening. Following the title and abstract review, 27 studies were excluded for not meeting the inclusion criteria. Eleven full-text articles were then assessed for eligibility. Of these, eight were excluded for the following reasons: three studies involved irrelevant comparisons not directly assessing the effectiveness of MINB versus IANB [20,22,23], four studies included inappropriate populations or interventions such as molars, extractions, or alternate anesthetic techniques [24-27] , and one study was classified as non-original research [28]. Ultimately, three randomized controlled trials met all eligibility criteria and were included in the qualitative and quantitative synthesis (Figure 1) [18,19,29].

Study Characteristics

This review included three RCTs published between 2016 and 2025, conducted in Turkey, Iran, and India. All studies evaluated the anesthetic efficacy of the MINB compared to the IANB in patients with SIP affecting mandibular premolars. The total sample comprised 287 participants, with a mean age ranging from 33 to 39 years. Female participants were slightly more represented in two of the three studies.

All trials evaluated pulpal anesthesia success after administering 1.8 mL of anesthetic solution used 4% articaine with 1:100,000 epinephrine, while one used 2% lidocaine with 1:200,000 epinephrine. Pain was assessed using either the Heft Parker Visual Analogue Scale (HP-VAS) or the Numeric Rating Scale (NRS), with outcomes defined as pulpal anesthesia success during endodontic access.

The distribution of first and second premolars treated was relatively balanced across studies. One study reported that combining MINB with IANB significantly improved anesthetic success rates [18]. Ghabraei et al. found comparable efficacy between the two techniques when using articaine [19]. In contrast, Sülek et al. (2025) also observed similar outcomes overall, but noted that MINB was less effective in second premolars compared to first premolars [29] . The main characteristics of the included studies are summarized in Table 1.

**Table 1:** Characteristics of included studies. IANB: Inferior alveolar nerve block; MINB: mental/incisive nerve block

Author (Year)	Country	Study Design	Sample Size	Mean Age	Sex	Treated Teeth Type	Pulpal Diagnosis	Interventions	Outcome Measure	Definition of Pulpal Anesthesia Success	Anesthetic Solution	Anesthetic Volume	Pain Scale	Results
Sülek et al. (2025) [29]	Turkey	RCT	120	39	Male: 43.3% Female: 56.6%	First Premolars: 45% Second Premolars: 55%	SIP	Group 1: MINB technique; Group 2: IANB technique	Pulpal Anesthesia	Defined as patients completing treatment with no pain (NRS = 0) or mild pain (NRS ≤ 3) without additional anesthesia.	4% articaine with 1:100,000 epinephrine	1.8 ml	NRS	Both MINB and IANB offer similar levels of pain management for mandibular premolars during endodontic treatment; however, MINB was less effective when used on second premolars compared to first premolars
Ghabraei et al. (2019) [19]	Iran	RCT	64	36	Male: 44% Female: 56%	First Premolars:48% Second Premolars:52%	SIP	Group 1: MINB technique Group 2: IANB technique	Pulpal Anesthesia	Determined by the proportion of patients who experienced no pain or only mild discomfort throughout the procedure	4% articaine with 1:100,000 epinephrine	1.8 ml	HP-VAS	MINB and IANB with 4% articaine demonstrated comparable effectiveness in achieving anesthesia of mandibular premolars affected by irreversible pulpitis.”
Aggarwal et al. (2016) [18]	India	RCT	103	33	Male: 53% Female: 47%	First Premolars:44% Second Premolars:56%	SIP	Group 1: MINB technique Group 2: IANB technique Group 3: IANB + MINB technique	Pulpal Anesthesia	Defined as the absence of pain or only mild discomfort during endodontic procedures, as measured by an HP VAS score below 55 mm.	2% lidocaine with 1:200,000 epinephrine	1.8 ml	HP-VAS	MINB and IANB showed similar anesthetic effectiveness, and their combination significantly increased the anesthetic success rate.

Risk of Bias Assessment and Certainty of Evidence

**Figure 2:**
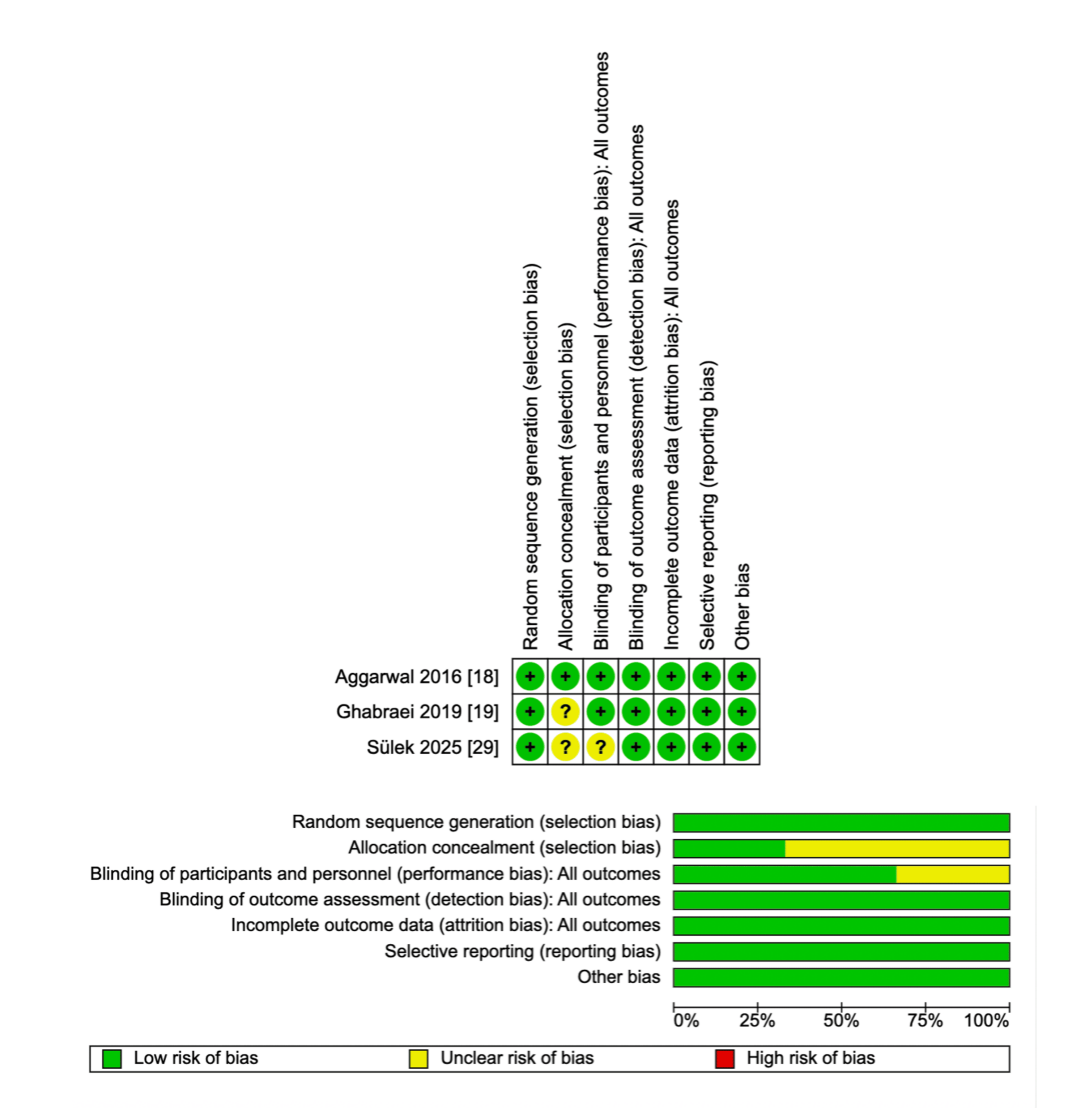
Risk of bias assessment

The assessment revealed that while one study [18] had a low risk of bias across all domains, the other two trials [19,29] presented unclear risk in key areas, particularly regarding allocation concealment and blinding of participants and personnel (Figure 2).

According to the GRADE approach, the certainty of evidence for the primary outcome was rated as low, mainly due to methodological limitations, indirectness, and imprecision. Two of the three RCTs showed unclear allocation concealment and lack of blinding, while in two studies the mental nerve block was performed without explicit foramen localization, reducing directness. Additionally, wide CIs crossing the null value indicated imprecision. Inconsistency was not considered serious (I² = 0%), and publication bias could not be assessed due to the limited number of studies (Table 2).

**Table 2:** GRADE evidence profile IANB: Inferior alveolar nerve block; MINB: mental/incisive nerve block; SIP: symptomatic irreversible pulpitis

Certainty Assessment							Summary of Findings				
Participants (studies) Follow-up	Risk of Bias	Inconsistency	Indirectness	Imprecision	Publication Bias	Overall Certainty of Evidence	Study Event Rates (%)		Relative effect (95% CI)	Anticipated Absolute Effects	
							With Favours IANB	With Favours MINB		Risk with Favours IANB	Risk difference with Favours MINB
Anesthetic Efficacy of MINB vs IANB on Mandibular Premolars with SIP											
286 (3 RCTs)	Serious	Not serious	Serious^a^	Serious^b^	None	⨁◯◯◯ Very low^a,b^	92/143 (64.3%)	99/143 (69.2%)	OR 1.25 (0.74 to 2.12)	92/143 (64.3%)	49 more per 1000 (from 72 fewer to 149 more)

Primary and Secondary Outcomes

**Figure 3:**
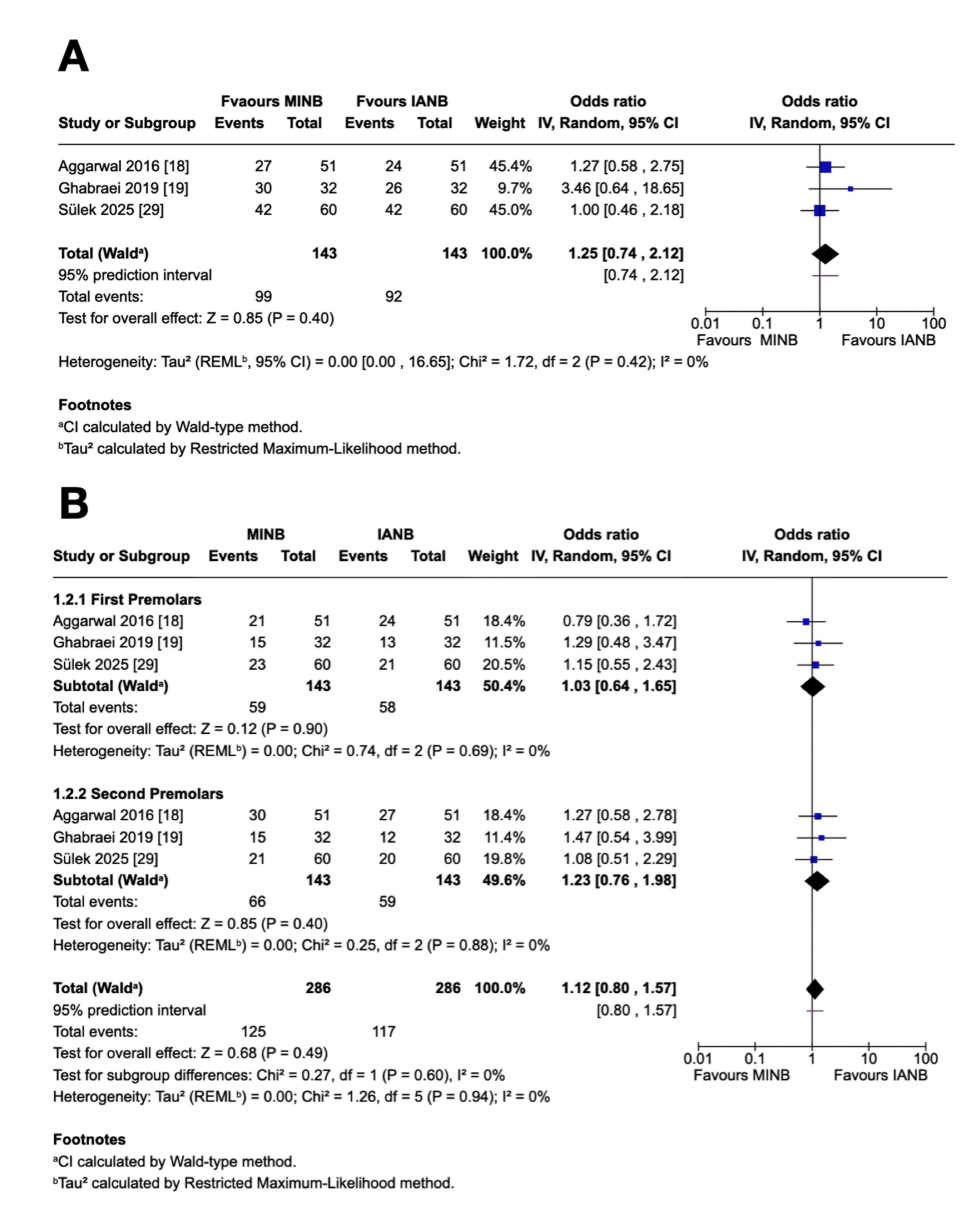
Forest plots comparing the anesthetic success of the MINB versus the inferior IANB in mandibular premolars with SIP. A: Overall comparison of anesthetic success rates across three randomized controlled trials. B: Subgroup analysis based on the premolar location. IANB: Inferior alveolar nerve block; MINB: mental/incisive nerve block; SIP: symptomatic irreversible pulpitis

Three RCTs comparing the anesthetic success of the MINB and IANB in mandibular premolars diagnosed with SIP [18,19,29] were included in the meta-analysis. The overall pooled analysis showed no statistically significant difference in success rates between the MINB and IANB techniques (OR = 1.25; 95% CI: 0.74 to 2.12; P = 0.40), with low heterogeneity (I² = 0%). The 95% PI was 0.74-2.12, indicating that the true effect in a future comparable study could plausibly range from favoring IANB to favoring MINB, consistent with no reliable difference in efficacy (Figure 3A).

Two trials reported safety outcomes and no serious adverse events occurred. In the study by Ghabraei et al., minor local reactions were noted with the MINB, slight subjective swelling in three participants and tenderness to palpation at the injection site in one, while one participant in the IANB group experienced prolonged numbness that resolved within 48 [19]. In the study by Sülek et al., adverse events were infrequent and transient: in the IANB group, temporary facial nerve palsy, pain on mouth opening, a palpable masseter, and slight swelling were observed; in the MINB group, slight swelling and mild pain were reported; no patient required rehospitalization [29]. Aggarwal et al. did not report safety outcomes [18].

Subgroup analysis based on the type of premolar treated revealed that for first premolars, the combined results showed no significant difference between techniques (OR = 1.03; 95% CI: 0.64-1.65; P = 0.90; I² = 0%; 95% PI: 0.64-1.65). Similarly, for second premolars, no significant difference was found (OR = 1.23; 95% CI: 0.76-1.98; P = 0.40; I² = 0%; 95% PI: 0.76-1.98). The test for subgroup differences was also not significant (Chi² = 0.27; P = 0.60), indicating that the effect of the anesthetic technique did not significantly vary between first and second premolars (Figure 3B).

**Figure 4:**
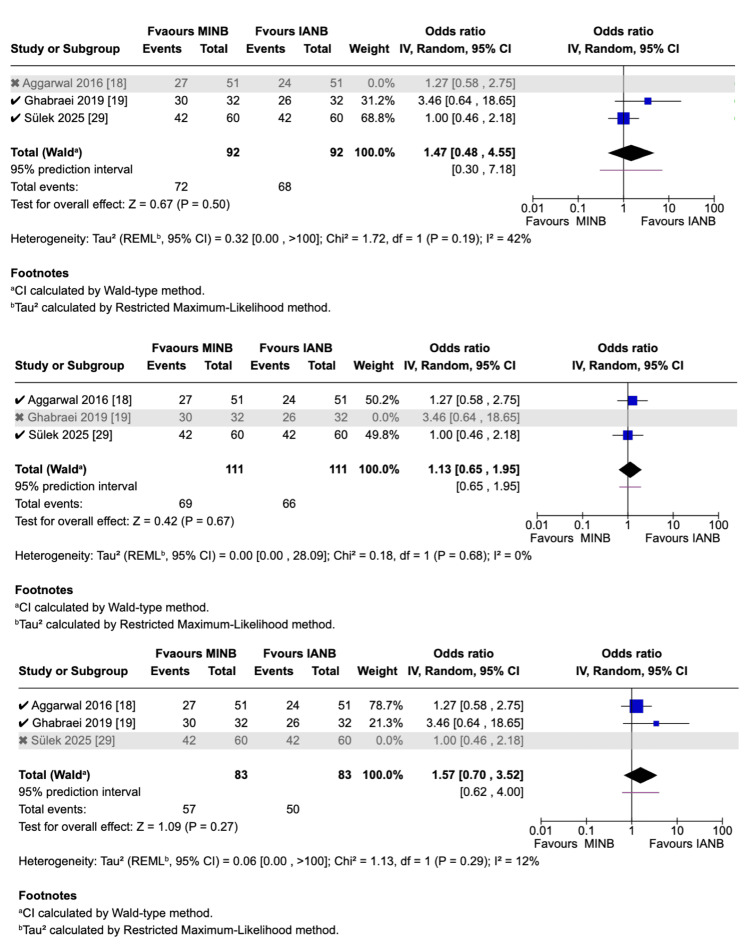
Leave-one-out sensitivity analysis.

Leave-one-out sensitivity analyses excluding Aggarwal (2016) or Ghabraei (2019) produced pooled odds ratios similar to the primary estimate with CIs crossing 1.0, whereas excluding Sülek (2025) yielded OR 1.57 (95% CI 0.70-3.52); overall conclusions remained unchanged (Figure 4 in the Appendices).

Discussion

Summary of Main Findings

This systematic review applied rigorous inclusion criteria and a comprehensive search strategy to identify methodologically sound RCTs, ensuring homogeneity across studies and minimizing selection bias. To our knowledge, this is the first meta-analysis focused on comparing the anesthetic success of MINB and IANB in mandibular premolars with SIP, incorporating only RCTs with standardized outcome assessments to enhance the validity and reliability of pooled estimates.

The clinical relevance of this meta-analysis lies not only in demonstrating that MINB and IANB yield similar anesthetic success rates in mandibular premolars with SIP but also in confirming the systematic need for supplemental anesthesia to achieve complete pulpal anesthesia. The consistent need for adjunctive techniques regardless of the initial block chosen emphasizes that neither MINB nor IANB alone can reliably achieve profound anesthesia in this clinical context.

Comparison With Previous Studies and Methodological Considerations

No significant differences in anesthetic efficacy were observed between IANB and MINB across the pooled data, consistent with the findings of the individual RCTs included in this review [18,19,29]. Both techniques appear to provide similar anesthetic outcomes for mandibular premolars with SIP. Notably, one study reported that combining MINB with IANB significantly improved success rates, reaching 82%, highlighting a potential synergistic effect in challenging clinical scenarios such as SIP [18].

The pre-specified technique subgroup (true MINB with mental localization vs injections without localization) was not estimable. Leave-one-out analyses suggest that technique heterogeneity matters: excluding the localized-foramen trial shifts the summary toward IANB but remains imprecise [29], whereas the localized-foramen trial alone indicates no difference. Together, this implies possible misclassification and reduced directness, supporting downgrading for indirectness and the need to standardize and explicitly report foramen localization in future trials

Two RCTs were identified but excluded from the quantitative synthesis because they did not directly compare MINB with IANB. Salem et al. (2025) evaluated the anesthetic efficacy of intraligamentary injection versus the MINB, while Srinivasan et al. (2017) compared buccal infiltration with the inferior alveolar nerve block specifically in mandibular second premolars [22,23]. Although these studies were not eligible for inclusion, acknowledging them provides context for the indirectness of the available evidence and highlights the limited number of trials directly assessing MINB efficacy in this specific population.

Importantly, none of the studies reported achieving complete pulpal anesthesia with either technique alone, even when both were combined, highlighting the consistent need for supplemental methods such as buccal infiltration or intraligamentary injection [18,19,29]. These findings are in line with the results of a recent systematic review and meta-analysis by Gupta et al., which demonstrated that the addition of intraligamentary injection significantly enhanced the anesthetic success rate in mandibular teeth with SIP, reinforcing the critical role of adjunctive techniques in overcoming the biological resistance of inflamed pulp tissue to conventional anesthesia [2].

This persistent need for supplemental anesthesia can be attributed to the underlying pathophysiology of SIP. Inflammatory processes in the pulp lead to the upregulation of tetrodotoxin-resistant sodium channels (Nav1.8 and Nav1.9) and TRPV1 receptors, which are less sensitive to conventional anesthetics such as lidocaine. Additionally, the inflamed environment results in local acidosis, increased vascular permeability, and peripheral sensitization, all impairing anesthetic diffusion and action, thereby reducing the likelihood of achieving profound and lasting pulpal anesthesia through standard techniques alone [30]. Subgroup analysis revealed no significant differences in anesthetic success between first and second premolars for either MINB or IANB, indicating that tooth type does not notably affect efficacy in SIP cases. This may relate, in the case of MINB, to anatomical variability in the MF position, typically near the second premolar apex but sometimes between apices or closer to the first, allowing effective anesthetic diffusion regardless of tooth [7,31,32]. For IANB, deposition near the mandibular nerve before branching ensures both premolars receive consistent anesthesia [33]. 

An important methodological point concerns the injection approach in the included studies. Only Sülek et al. localized the MF before anesthetic deposition, consistent with the classical MINB technique [29]. In contrast, Ghabraei et al. and Aggarwal et al. injected into the buccal vestibule without MF localization, likely representing buccal infiltrations rather than true MINB [18,19]. This variation may affect result interpretation, as the reported success could reflect infiltration outcomes. Future trials should standardize MF localization to ensure accurate assessment of MINB efficacy.

The width of the CIs observed across studies reflects the limited number and sample size of available RCTs. Although pooled estimates favored no significant difference between techniques, the wide CIs suggest statistical imprecision and uncertainty regarding the true effect size. This observation, together with the low heterogeneity (I² = 0%), supports the GRADE assessment of low certainty and underscores the need for future trials with larger, standardized populations to enhance estimate precision.

Patient-Centered Outcomes

Regarding patient comfort and injection-related pain, Ghabraei et al. reported that the MINB elicited significantly less pain during injection than the IANB, along with a faster onset of anesthesia [19]. These findings are consistent with the results of Kaufman et al., who identified IANB as the most painful technique among various intraoral anesthesia methods. In contrast, the MINB was perceived as less uncomfortable due to its more superficial and simplified administration [34]. This consistency in patient perception suggests that MINB may be a more comfortable alternative during the injection phase, which could enhance patient acceptance and cooperation.

In terms of onset time, MINB consistently demonstrated a faster initiation of pulpal anesthesia compared to IANB across several studies. Ghabraei et al. reported that MINB achieved anesthesia more rapidly than IANB, a finding corroborated by Sulek et al., who observed that the latency period for MINB ranged between 2 and 5 minutes, while IANB required between 4 and 9 minutes to reach clinical efficacy [19,29]. This difference may be attributed to the more superficial deposition of the anesthetic in MINB and its proximity to terminal nerve branches, which facilitates quicker diffusion and nerve blockade [35]. In clinical settings, a shorter onset time can translate into more efficient workflow and reduced patient anxiety, particularly in cases of SIP where time-sensitive pain control is critical.

MINB was associated with slightly higher post-injection discomfort during the first few days, as Ghabraei et al. reported significantly greater residual pain in the MINB group compared to IANB [19]. Nevertheless, pain levels remained within the mild range and subsided steadily over time. This early discomfort may be attributed to localized tissue irritation around the MF, especially in inflamed pulps. Furthermore, no cases of paresthesia have been reported with MINB across multiple studies, while IANB has occasionally been associated with prolonged lip or tongue numbness [36,37].

Articaine is widely used in dental practice due to its superior tissue diffusion and rapid onset. Although concerns have been raised in the past regarding potential neurotoxicity, particularly in mandibular block techniques, recent systematic reviews and meta-analyses support its safety when used properly. Evidence indicates that adverse events are rare and often associated with improper technique or excessive dosing [26]. Therefore, when administered below neurotoxic thresholds and avoiding intraneural injection, articaine remains a safe and effective option for local anesthesia, especially for buccal infiltrations in mandibular premolars [16].

Limitations

This review has certain limitations that should be acknowledged. The evidence base is narrow: with only three randomized trials, serious risk of bias, and variability in how MINB was performed (e.g., inconsistent MF localization), this meta-analysis should be interpreted with caution and cannot provide definitive clinical guidance. The exclusive inclusion of patients with SIP restricts generalizability to healthy individuals or those with other dental pathologies. Reliance on patient-reported pain scales may introduce response variability. Although anesthetic volume and injection duration were standardized, residual differences may still influence anesthetic efficacy. Given the small evidence base, subgroup analyses (first vs second premolars; articaine vs lidocaine) were underpowered and treated as exploratory. Finally, the small total sample and the exclusion of alternative techniques (e.g., intraosseous or intraligamentary injections) may reduce external validity and should guide the design of future higher-quality trials.

## Conclusions

This meta-analysis of three RCTs found comparable anesthetic success rates between MINB and IANB in mandibular premolars with SIP, based on low-certainty evidence. Although both techniques demonstrated similar efficacy, achieving complete pulpal anesthesia frequently required supplemental anesthesia. These findings suggest that MINB can be considered a viable alternative to IANB in clinical scenarios involving SIP. Future well-designed RCTs with standardized methodologies are warranted to strengthen the current evidence base.

## Appendices

**Table 3:** Search strategy.

Database	Search Strategy	Search Results
Pubmed	(“Mental Nerve Block”[Mesh] OR “Mental/Incisive Nerve Block”[tiab]) AND (“Inferior Alveolar Nerve Block”[Mesh] OR [tiab]) AND (“Pulpitis”[Mesh] OR “Symptomatic Irreversible Pulpitis”[tiab] OR “Irreversible Pulpitis”[tiab] OR “Pulpitis”[tiab] OR “Symptomatic”[tiab]) AND (“Premolar”[Mesh] OR “Mandibular Premolars”[tiab])	6
Web of Science	TS=("Mental Nerve Block" OR "Mental/Incisive Nerve Block") AND TS=("Inferior Alveolar Nerve Block") AND TS=("Symptomatic Irreversible Pulpitis" OR "Irreversible Pulpitis" OR "Pulpitis" OR "Symptomatic") AND TS="Mandibular	4
Science Direct	("Mental Nerve Block" OR "Mental Incisive Nerve Block") AND ("Inferior Alveolar Nerve Block") AND ("Symptomatic Irreversible Pulpitis" OR "Irreversible Pulpitis" OR "Pulpitis" OR "Symptomatic") AND ("Premolar" OR "Mandibular Premolars")	9
Cochrane Database	("Mental Nerve Block" OR "Mental Incisive Nerve Block") AND ("Inferior Alveolar Nerve Block") AND ("Symptomatic Irreversible Pulpitis" OR "Irreversible Pulpitis" OR "Pulpitis" OR "Symptomatic") AND ("Premolar" OR "Mandibular Premolars")	8
Google Scholar	("Mental Nerve Block" OR "Mental/Incisive Nerve Block") AND ("Inferior Alveolar Nerve Block") AND ("Symptomatic Irreversible Pulpitis" OR "Irreversible Pulpitis" OR "Pulpitis" OR "Symptomatic") AND ("Mandibular Premolars")	25

**Table 4:** Data extraction.

Study (year)	Country	Anesthetic solution	MINB (events/total)	IANB (events/total)	Odds Ratio (95% CI)	Weight (%)						
Aggarwal et al., 2016	India	4% Articaine + 1:100,000 epinephrine	27 / 51	24 / 51	1.27 [0.58 – 2.75]	45,4						
Ghabraei et al., 2019	Iran	4% Articaine + 1:100,000 epinephrine	30 / 32	26 / 32	3.46 [0.64 – 18.65]	9,7						
Sülek et al., 2025	Turkey	4% Articaine + 1:100,000 epinephrine	42 / 60	42 / 60	1.00 [0.46 – 2.18]	45						
Pooled estimate (random-effects, REML)	—	—	99 / 143	92 / 143	1.25 [0.74 – 2.12]	100						
Subgroup	Studies (n)	Events MINB	Total MINB	Events IANB	Total IANB	Pooled OR	CI 95% (lower)	CI 95% (upper)	Heterogeneity I² (%)	Test for overall effect (Z, P)	Prediction interval (95%) - lower	Prediction interval (95%) - upper
First premolars	3	59	143	58	143	1,03	0,64	1,65	0	Z=0.12, P=0.90		
Second premolars	3	66	143	59	143	1,23	0,76	1,98	0	Z=0.85, P=0.40		
Overall (tooth-type analysis)	6	125	286	117	286	1,12	0,8	1,57	0	Z=0.68, P=0.49	0,8	1,57

**Table 5:** Screening logs

Stage	Database	Reference	Title (First Author, Year)	Decision	Reason for Exclusion			Database	Records Retrieved	Articles Retrieved	Duplicates Removed	Records Screened	Full-text Assessed	Excluded (Reasons)	Included in Meta-analysis
Title/Abstract Screening	PubMed	22	Dias-Junior et al., 2021	Excluded	Compared several anesthetic methods for mandibular posterior teeth, not limited to MINB vs IANB. The study design did not isolate the specific comparison of interest.			PubMed	6	Aggarwal et al., 2016; de Almeida Barros Mourão et al., 2020; Ghabraei et al., 2019; Hayes, 2025; Nagendrabab et al., 2019; Sülek et al., 2025	38	38	12	8 excluded (3 irrelevant comparisons , 4 inappropriate, 1 non-original)	3
Title/Abstract Screening	PubMed	23	Salem et al., 2025	Excluded	Evaluated intraligamentary injection versus incisive nerve block; not directly comparable to IANB or MINB in the context of endodontic anesthesia.			Web of Science	4	Aggarwal et al., 2016; Ghabraei et al., 2019; Nagendrabab et al., 2019; Sülek et al., 2025					
Title/Abstract Screening	PubMed	24	Srinivasan et al., 2017	Excluded	Compared buccal infiltration and IANB in mandibular second premolars, but did not include MINB as an intervention arm.			ScienceDirect	9	Sülek et al., 2025; Nagendrabab et al., 2019; Aggarwal et al., 2016; Zanjir et al., 2019; Brigandiello-Petersen, 2020; Kumar et al., 2020; Drossman et al., 2013; Majid et al., 2019; Shurtz, 2011					
Full-Text Eligibility	ScienceDirect	25	Dressman et al., 2013	Excluded	Evaluated primary and repeat articaine infiltrations at the mental nerve region in healthy volunteers, not in patients with symptomatic irreversible pulpitis (SIP).			Cochrane Database	8	Aggarwal et al., 2016; de Almeida Barros Mourão et al., 2020; Ghabraei et al., 2019; Hayes, 2025; Nagendrabab et al., 2019; Sülek et al., 2025; Dias-Junior et al., 2010; Dossman et al., 2013					
Full-Text Eligibility	Cochrane	26	Kumar et al., 2020	Excluded	Investigated bilateral versus unilateral MINB techniques during management of mandibular incisors, not premolars, and lacked an IANB control group.			Google Scholar	25	Aggarwal et al., 2016; de Almeida Barros Mourão et al., 2020; Ghabraei et al., 2019; Hayes, 2025; Nagendrabab et al., 2019; Sülek et al., 2025; Brigandiello-Petersen, 2020; Zanjir et al., 2019; Kumar et al., 2020; Majid et al., 2019; Paikroh et al., 2024; Van Meter et al., 2013; Karamkar et al., 2021; and others (n=25 reported)					
Full-Text Eligibility	PubMed	27	Samiei et al., 2020	Excluded	Focused on anesthetic efficacy of MINB for first molars with asymptomatic irreversible pulpitis; tooth type and clinical condition did not meet inclusion criteria.										
Full-Text Eligibility	Google Scholar	28	Siddique et al., 2025	Excluded	Assessed anesthetic efficacy of MINB for extractions rather than endodontic treatment; procedure type not aligned with review objectives.										
Full-Text Eligibility	Cochrane	29	Hayes, 2025	Excluded	Non-original publication (evidence summary/commentary) that did not provide extractable clinical data for meta-analysis.										
